# Comparison of effect between dartos fascia and tunica vaginalis fascia in TIP urethroplasty: a meta-analysis of comparative studies

**DOI:** 10.1186/s12894-020-00737-9

**Published:** 2020-10-15

**Authors:** Hao Yang, Xiao-xiao Xuan, Dong-lai Hu, Hang Zhang, Qiang Shu, Xiao-dong Guo, Jun-fen Fu

**Affiliations:** 1 Zhejiang University School of Medicine, Hangzhou, China; 2 Children’s Hospital, Zhejiang University School of Medicine, Hangzhou, China; 3 National Clinical Research Center for Child Health, Hangzhou, China; 4 Department of Pediatric Surgery, Zhejiang University Jinhua Hospital, Jinhua, China

**Keywords:** Hypospadias, Urethrocutaneous fistula, Dartos fascia, Tunica vaginalis fascia, Meta-analysis

## Abstract

**Background:**

Tubularized incised plate (TIP) urethroplasty is the most commonly performed procedure for hypospadias. Several flap procedures have been recommended to decrease the postoperative complication rate in TIP repair, but no single flap procedure is ideal. This study aimed to compare the outcomes of dartos fascia (DF) and tunica vaginalis fascia (TVF) as intermediate layers in TIP urethroplasty.

**Methods:**

We searched PubMed, EMBASE, the Cochrane Library, Web of Science, clinicaltrials.gov, and other sources for comparative studies up to April 16, 2020. Studies were selected by the predesigned inclusion criteria. The primary outcomes were postoperative complications. The secondary outcomes were functional and cosmetic outcomes.

**Results:**

The pooled RR with 95% CI were calculated. We extracted the relevant information from the included studies. Only 6 comparative studies were included. No secondary outcomes were reported. The RR of the total complications rate for DF was 2.41 (95% CI 1.42–4.07, *P* = 0.0001) compared with TVF in TIP repair. For each postoperative complication, the RRs were 6.48 (2.20–19.12, *P* = 0.0007), 5.95 (1.13–31.30, *P* = 0.04), 0.62 (0.25–1.52, *P* = 0.29), and 0.75 (0.23–2.46, *P* = 0.64) for urethrocutaneous fistula, prepuce-related complications, meatal/urethral stenosis, and wound-related complications, respectively.

**Conclusions:**

This meta-analysis reveals that compared to DF, TVF is a better option in TIP repair in terms of decreasing the incidence of the total postoperative complications, urethrocutaneous fistula, and prepuce-related complications. However there is limited evidence for functional and cosmetic outcomes. Overall, larger prospective studies and long-term follow-up data are required to further demonstrate the superiority of TVF over DF.

**Trial registration:**

PROSPERO CRD42019148554.

## Background

Hypospadias, resulting from the disruption of the normal urethral formation, is one of the most common congenital malformations in male infants, with an overall predicted prevalence of 20.9 cases per 10,000 male live births around the world [1]. The international total prevalence increased 1.6 times from 1980–2010, by 0.25 cases per 10,000 births per year [1]. The current purpose of hypospadias surgery is to improve the functional and cosmetic outcomes while minimizing the incidence of postoperative complications and avoiding reoperations. The tubularized incised plate (TIP) urethroplasty is the most commonly performed procedure for hypospadias [2]. Additional urethral coverage is now routinely used by most paediatric urologists to reduce the incidence of postoperative complications [3]. Dartos fascia (DF) and tunica vaginalis fascia (TVF) are the two most widely used urethral coverages with good surgical results while results reported by different studies vary [4–6]. At present, there has been no consensus on the better choice between the DF and TVF flap techniques or on the short- and long-term outcomes of both techniques [3]. This meta-analysis aimed to compare the outcomes of TVF and DF as intermediate layers in TIP hypospadias repair.

## Methods

### Registration

This study is registered with PROSPERO, registration number CRD42019148554. The completed PRISMA checklist for NMA was presented in the Appendices (Additional file 1: Table S1).

### Search strategies

Four electronic databases were searched up to April 16, 2020, including PubMed, EMBASE, Cochrane Central and Web of Science. The searching strategy in PubMed was presented as follows: ((((((((hypospadias repair[Title/Abstract]) OR hypospadias surgery[Title/Abstract]) OR tubularized incised plate[Title/Abstract]) OR TIP[Title/Abstract]) OR tubularised incised plate[Title/Abstract]) OR Snodgrass repair[Title/Abstract]))) AND (((((((urethral cover*[Title/Abstract]) OR soft tissue cover*[Title/Abstract]) OR additional cover*[Title/Abstract]) OR subcutaneous flap*[Title/Abstract]) OR flap cover*[Title/Abstract]) OR dartos[Title/Abstract]) OR tunica vaginalis[Title/Abstract]). We also screened clinicaltrials.gov, some international professional conference abstract (such as the Society for Pediatric Urology, European Society for Paediatric Urology, Asia–Pacific Association of Pediatric Urologists, American Pediatric Surgical Association and World Federation of Associations of Pediatric Surgery) and the references of included articles and published reviews to identify additional relevant publications.

### Inclusion criteria

The studies were selected according to the following criteria: (1) participants were diagnosed with primary hypospadias and received TIP urethroplasty, (2) the study compared TV with DVF, (3) the study reported the incidence of postoperative complications with/without functional or cosmetic outcomes, (4) the article was written in English, and (5) the study was a randomized controlled trial (RCT), quasi-RCT, non-RCT, retrospective, prospective, or concurrent cohort study. Studies with the full text unavailable, ineligibile study types (single-arm trials, reviews, expert opinions, comments, letters to the editor, case report/series, studies on animals and conference reports), or no outcome of interest reported were excluded. Reoperations, non-TIP repair, fistula repair, patients with severe chordee that required UP excision or two-stage repair were also excluded. Complications were defined as urethrocutaneous fistula, meatal/urethral stenosis, wound-related complications (wound dehiscence, wound infection, or meatal retraction), prepuce-related complications (prepuce fistula, prepuce dehiscence, skin necrosis, or phimosis), testis-related complications (testicular ascent, torsion, infection, or atrophy), and urinary tract infection (UTI). Functional outcomes included urinary stream direction, maximum flow rate, voided volume, average flow rate, voiding time, flow curve shape, and residual urine. Cosmetic outcomes included HOSE (hypospadias objective scoring evaluation) score [7], PPPS (Pediatric Penile Perception Score) [8], and HOPE (Hypospadias Objective Penile Evaluation) score [9]. The primary outcomes were postoperative complications. The secondary outcomes were functional and cosmetic outcomes.

### Data extraction and quality assessment

Two independent authors (HY, QS) screened all retrieved titles and abstracts according to the pre-described inclusion criteria to identify potentially eligible studies. After screening, we accessed the full text to determine the final included studies independently. The following data from each included study were extracted: study characteristics (first author, published year, study design, hypospadias type, surgery type, and follow-up time) and patient characteristics (patient numbers, patient age, incidence of each complication, evaluation methods and corresponding values of functional and cosmetic outcomes). For the whole process, discrepancies were resolved by discussion between the two reviewers and re-evaluation with a third author (XG). The methodological quality of all included studies were independently assessed using the 27-item Downs and Black scale [10], a validated tool for evaluating randomized and nonrandomized studies, which consists of 4 aspects: study reporting, external validity, internal validity, and the power of the study, and the total scores ranged from 0 to 32. We used the following criteria to assess the risk of bias of study: low-risk study (total score ranged from 24 to 32), moderate-risk study (total score ranged from 16 to 23) and high-risk study (total score ranged from 0 to 15). Interrater consistency for the Downs and Black score assessment was analyzed using the Kappa statistic, in which the power of the Kappa value was interpreted as poor (Kappa value ≤ 0.40), good (Kappa value ranged from 0.40–0.75), and excellent (Kappa value > 0.75) agreement.

### Statistical analysis

We used RevMan 5.3 (Cochrane Library, Oxford, UK) and STATA 13.1 (Corp LP, College Station, TX, USA) software programs to perform this meta-analysis and relevant subgroup analyses. Kappa statistic was performed using IBM SPSS Statistics 23.0 software (IBM Corp., Armonk, NY, USA). The standardized mean difference (SMD) was calculated as the effect size for continuous outcomes and the risk ratio (RR) was calculated for dichotomous outcomes, both with 95% confidence intervals (CI). The Mantel–Haenszel method was used to calculate pooled RR [11]. Heterogeneity was evaluated by the Cochrane’s Q-statistic and the I^2^ statistic [12]. A random-effects model was used if heterogeneity was significant (the Q statistic was significant or I^2^ values > 50%). Otherwise, a fixed-effects model would be used. We used the funnel plot and Egger’s test to detect publication bias if at least 10 studies were available [12, 13]. Sensitivity and subgroup analyses were performed to assess the robustness of the results and to find the possible sources of heterogeneity. Several subgroup analyses were conducted, including different hypospadias types, study design, and other available groups. A *P* value < 0.05 was considered to be statistically significant.

## Results

### Literature selection and study characteristics

**Fig. 1:**
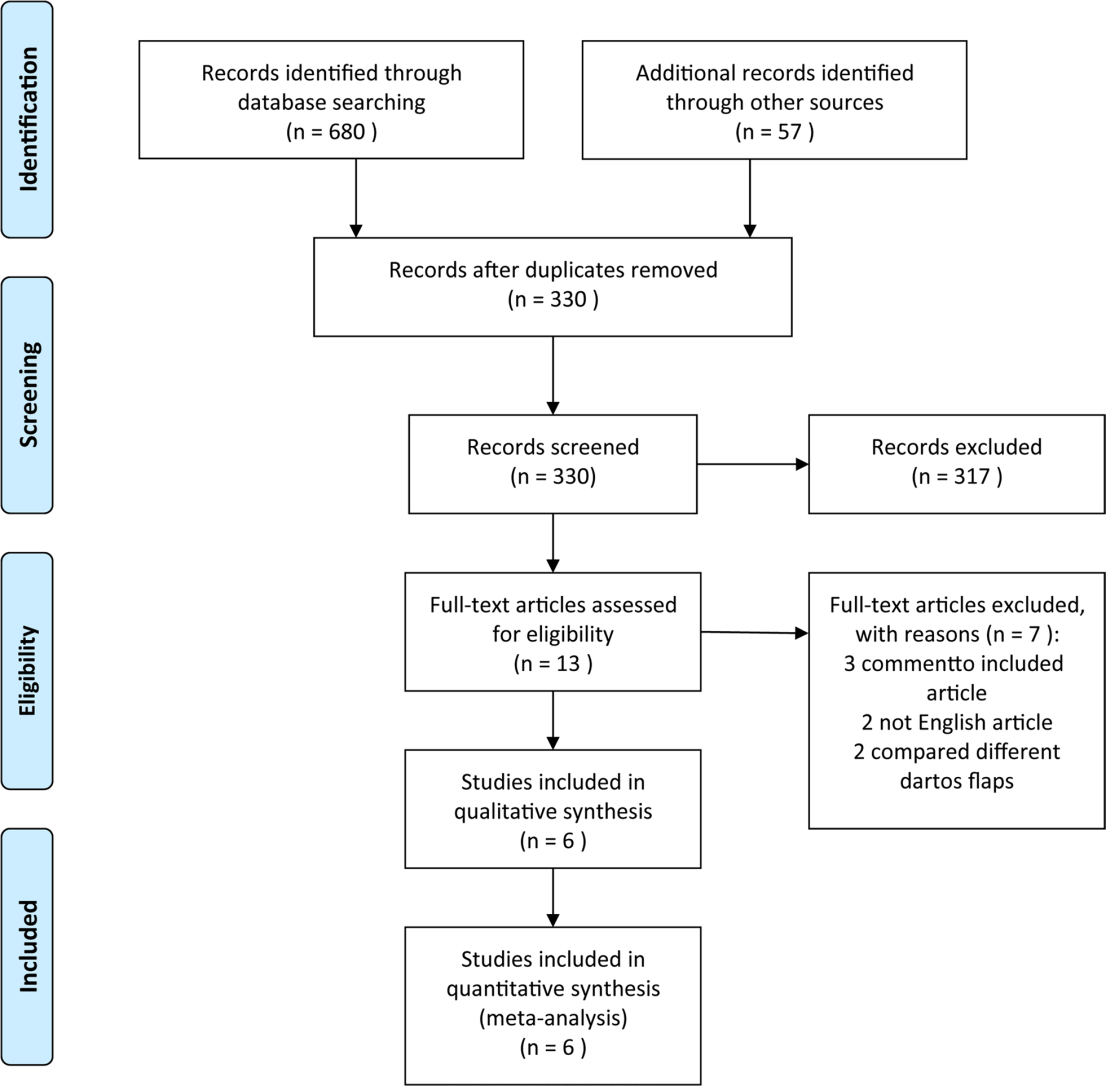
Flow diagram of study identification and screening for final inclusion

Of 680 database article titles and 57 trial register titles screened, 330 abstracts were reviewed, 13 full texts were reviewed, and 6 articles [14–19] met our inclusion criteria and were included in our meta-analysis (Fig. 1). The 6 comparative studies involved a total of 353 hypospadias patients (145 patients in the TVF group and 208 in the DF group). One study [15] was retrospective, and 5 [14, 16–19] were prospective. Two studies included distal and midshaft hypospadias [14, 18], and 4 [15–17, 19] included all types of hypospadias. All studies performed a one-stage TIP urethroplasty for primary hypospadias (Table 1). Of the included studies, 71/353 (20.11%) reported a postoperative complication: 34/353 (9.63) reported urethrocutaneous fistula, 15/353 (4.25) meatal/urethral stenosis, 12/353 (3.40%) prepuce-related complications, and 9/353 (2.55%) wound-related complications. Only 1 children had a mild testis torsion in DF group, and no instances of damage to testicular vessels, vas deferens, scrotal abscess or haematoma were reported in the included studies (Table 2).

**Table 1:** Summarizes the primary characteristics of the included trials

Author, year	Study design	Hypospadias type	Total number of patients	Mean age (range) (year)	Follow-up (mo)	Perioperative use of antibiotics	Postoperative complications	Functional and cosmetic outcomes
Babu 2013 [14]	Pro	Distal and midshaft	83	1.06 (0.75–1.6)	12	Intravenous + oral antibiotics	DF: 11 urethrocutaneous fistula, 4 meatal stenosis, 6 skin necrosis, 2 glans dehiscence TVF: 1 urethrocutaneous fistula, 1 glans dehiscence, 4 meatal stenosis	NA
Basavaraju 2017 [15]	Retro	Distal, midshaft and proximal	83	3.44 (1.6–12)	6–36	Intravenous + oral antibiotics	DF: 11 urethrocutaneous fistula, 1 meatal stenosis, 3 glans dehiscence TVF: 0	NA
Chatterjee 2004 [16]	Pro	Distal, midshaft and proximal	49	4.6 (1–22)	12–48(mean 24)	No	DF: 3 urethrocutaneous fistula TVF: 1 wound dehiscence	NA
Dhua 2012 [17]	Pro	Distal, midshaft and proximal	50	3.21 (1–12)	NA	Intravenous + oral antibiotics	DF: 3 urethrocutaneous fistula, 3 skin necrosis TVF: 1 wound dehiscence	NA
Gajbhiye 2018 [18]	Pro	Distal and midshaft	48	1.75 (1.5–4.33)	6–18	Intravenous + oral antibiotics	DF: 3 urethrocutaneous fistula, 3 skin necrosis TVF: 1 wound dehiscence	NA
Kurbet 2018 [19]	Pro	Distal, midshaft and proximal	40	4.85 (0.75–18)	> 6	No	DF: 2 urethrocutaneous fistula, 2 meatal stenosis, 1 urethral stricture TVF: 2 meatal stenosis, 1 urethral stricture, 1 mild testis torsion	NA

*Pro* prospective, *Retro* retrospective, *TIP* tubularized incised plate, *TVF* tunica vaginalis fascia, *DF* dartos fascia, *NA* not available

**Table 2:** Complications of TIP hypospadias repair in all included studies (*N* = 353)

Complication	n (%)	n/*N*
Urethrocutaneous fistula	34 (47.89)	9.63%
Meatal/urethral stenosis	15 (21.13)	4.25%
Prepuce-related complications	12 (16.90)	3.40%
Wound-related complications	9 (12.68)	2.55%
Testis-related complications	1 (1.41)	0.28%
Urinary tract infection	0 (0)	0
Total complications	71 (100)	20.11%

*n* number of complication, *N* number of total cases

### Assessment of methodological quality

Because only 1 RCT, 2 non-RCTs (1 non-randomized trial and 1 controlled before-after trial) and 3 cohort studies were included, we used the Downs and Black scale [10] for assessing methodological quality instead of the Cochrane Collaboration tool for RCT, the NOS (Newcastle–Ottawa Scale) for cohort studies, and MINORS (Methodological Index for Nonrandomized Studies) for non-RCTs.

For the detailed Down and Black scale items and scores of 2 independent raters see Additional file 1: Table S2. The mean Down and Black scores ranged from 16 to 25.5. Strengths included study reporting and external validity. Weaknesses included internal validity and the power of the study. The Kappa value of the interrater consistency for the Downs and Black score assessment was 0.571 (*P* = 0.121), which means we are in good agreement.

### Meta-analysis

**Fig. 2:**
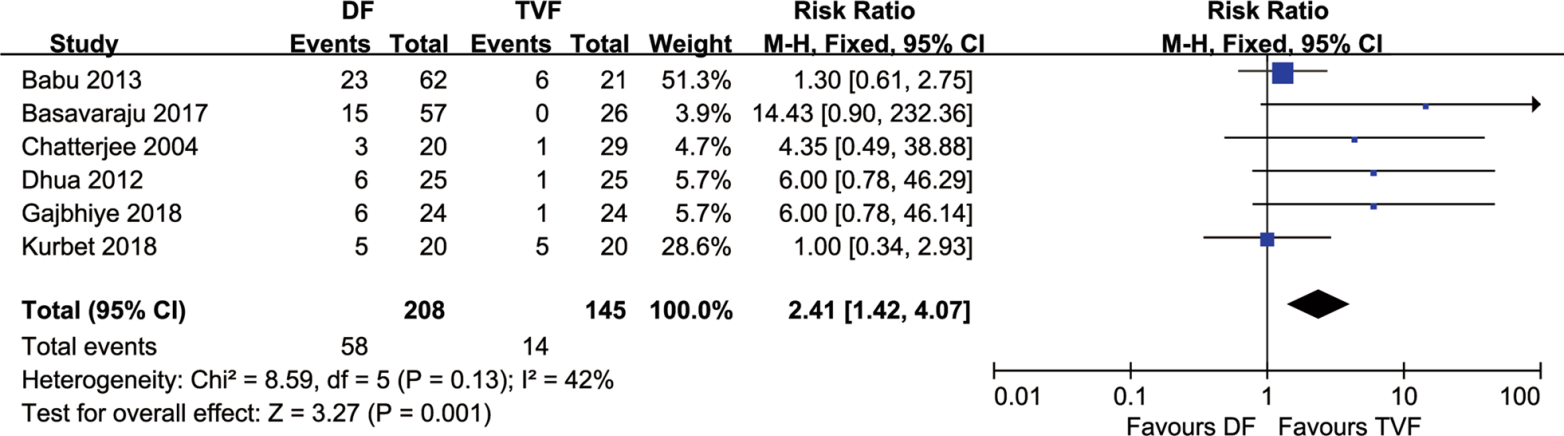
Forest plot of the comparisons the total complications between DF and TVF flap techniques. *DF* dartos fascia, *TVF* tunica vaginalis fascia

**Fig. 3:**
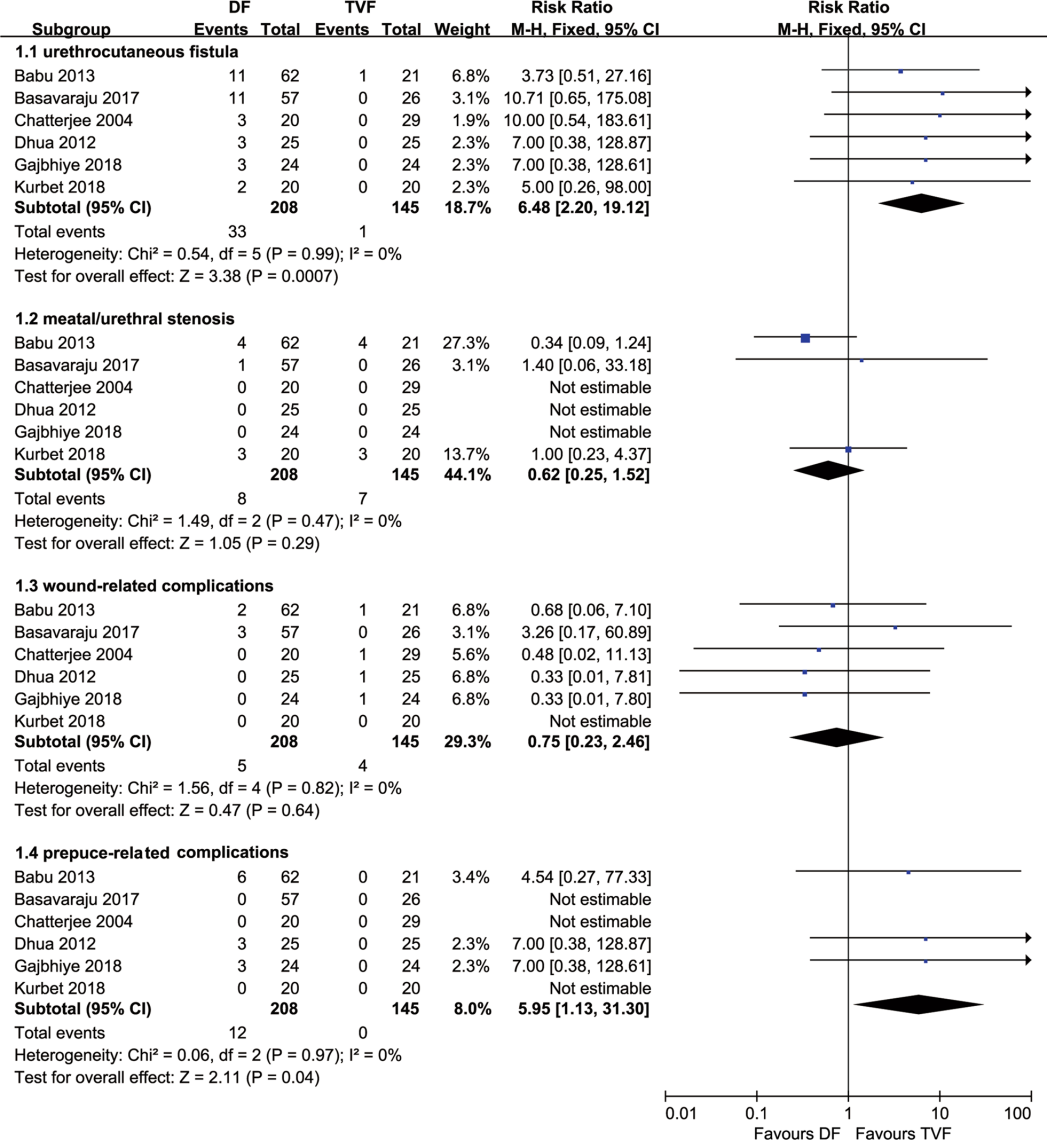
Forest plot of the comparisons each complication between DF and TVF flap techniques. *DF* dartos fascia, *TVF* tunica vaginalis fascia

A total of 6 comparative studies [14–19], comprising 353 hypospadias children with repaired TIP, were included. All studies reported the incidence of postoperative complications. The RR of the total complications rate for DF was 2.41 (95% CI 1.42–4.07, *P* = 0.0001) compared with TVF in TIP repair for hypospadias. The heterogeneity was not statistically significant (*P* = 0.13, *I*^2^ = 42%), and the fixed-effect model was used for analysis (Fig. 2). The RRs were 6.48 (2.20–19.12, *P* = 0.0007), 5.95 (1.13–31.30, *P* = 0.04), 0.62 (0.25–1.52, *P* = 0.29), and 0.75 (0.23–2.46, *P* = 0.64) for urethrocutaneous fistula, prepuce-related complications, meatal/urethral stenosis, and wound-related complications, respectively, when comparing DF to TVF (Fig. 3). The sources of heterogeneity were all statistically not significant (all *I*^2^ values were equal to 0%). No functional or cosmetic outcomes were reported in any of the included studies.

Since only 6 studies were included in this meta-analysis, we did not perform further analysis for publication bias. Sensitivity analyses were conducted by changing the model to a random-effect model and re-performing meta-analyses after omitting each study. The results of sensitivity analyses indicated that changing the model to a random-effect model and omitting any one study did not significantly influence our results, which stabilized the meta-analysis results (Additional file 1: Figs. S1 and S2). Subgroup analyses for postoperative complications of TIP between DF and TVF were conducted and the results are summarized in Table 3.

**Table 3:** Subgroup analyses for postoperative complications in meta-analysis

Subgroup	Total complications		Urethrocutaneous fistula		Meatal/urethral stenosisMeatal/urethral stenosis		Prepuce-related complications		Wound-related complications	
	RR (95% CI)	*P*	RR (95% CI)	*P*	RR (95% CI)	*P*	RR (95% CI)	*P*	RR (95% CI)	*P*
All patients	*2.41 (1.42–4.07)*		*6.48 (2.20–19.12)*		0.62 (0.25–1.52)		*5.95 (1.13–31.30)*		0.75 (0.23–2.46)	
Hypospadias type										
D & M	1.77 (0.89–3.53)	0.26	4.55 (0.90–23.05)	0.59	0.34 (0.09–1.24)	0.22	5.53 (0.73–41.82)	0.90	0.51 (0.08–3.27)	0.60
D, M & P	*3.25 (1.45–7.30)*		*8.32 (1.91–36.31)*		1.07 (0.28–4.10)		7.00 (0.38–128.87)		0.97 (0.20–4.63)	
Study design										
Retro	14.43 (0.90–232.26)	0.16	10.71 (0.65–175.08)	0.68	1.40 (0.06–33.28)	0.59	–	–	3.26 (0.17–60.89)	0.24
Pro	*1.92 (1.13–3.26)*		*5.63 (1.77–17.97)*		0.56 (0.22–1.43)		*5.95 (1.13–31.30)*		0.45 (0.11–1.90)	
Perioperative use of antibiotics										
Yes	*2.88 (1.51–5.46)*	0.24	*6.26 (1.76–22.22)*	0.90	0.45 (0.14–1.43)	0.40	–	–	0.82 (0.23–2.96)	0.76
No	1.47 (0.58–3.71)		7.26 (0.93–56.54)		1.00 (0.23–4.37)		*5.95 (1.13–31.30)*		0.48 (0.02–11.13)	

Statistically significant results are italicized

*Retro* retrospective, *Pro* prospective, *P* *p* value for interaction, *D, M & P* distal, midshaft and proximal

## Discussion

This meta-analysis was the first comprehensive synthesis of evidence for currently available comparisons between DF and TVF flap techniques for TIP urethroplasty in primary hypospadias patients in comparative studies. We included 6 comparative studies, comprising 353 hypospadias patients who received TIP repair with the use of DF or TVF flaps. Evidence of our findings came from the pooled estimate size for the primary outcomes, which showed that TVF was better than DF for the repair of hypospadias in terms of total postoperative complications, urethrocutaneous fistula, and wound-related complications. No significant difference was found in meatal/urethral stenosis and prepuce-related complications. No significant difference was found in different hypospadias type, study design, and perioperative use of antibiotics subgroups. Additional, sensitivity analyses verified the robustness of the results in this meta-analysis.

Reoperation for failed hypospadias or fistula repair has been considered a serious problem because the dense fibrotic tissue causes difficulties in wound healing and increases the rate of complications [20]. Over 300 surgical methods and modifications have been developed for repairing the hypospadias, while various complications have occurred, especially urethrocutaneous fistula, one of the most common complications of these techniques [21]. In our study, the incidence of urethrocutaneous fistula was 9.63%, followed by meatal/urethral stenosis (4.25%), consistent with the 7.50% (fistula) and 4.40% (stenosis) incidence rates in a systematic review [22]. Patient age, glans size, urethral defect length, urethral operation history, surgical procedure, type of surgical repair, chordee degree, magnification technique, caudal anesthesia, preoperative hormonal stimulation, and other many factors may relate to the development of complications postoperatively [21–25]. Additional soft coverages on the neourethra are also introduced to avoid these complications, especially to decrease the incidence of postoperative urethrocutaneous fistula. TVF, DF, Buck’s fascia, spongious tissue, external spermatic fascia, adipose tissue of the scrotum, adipose tissue of the spermatic cord, and a combination of tissues and platelet-rich plasma are used in different studies [26–32] with various outcomes. Among them, TVF and DF are the most popular flaps used in the repair of hypospadias and fistula.

Objective and comprehensive assessments of the outcome of hypospadias repair may have a major impact on future clinical practice. Evaluations of outcome after hypospadias repair include complication rate, cosmetic outcome, functional outcome, and even the effects on psychology. In this study, we focused on assessing the effect of DF and TVF on the outcomes of complication rate after TIP urethroplasty in primary hypospadias. For functional and cosmetic results in our meta-analysis, the conclusions are uncertain due to the limited evidence. The assessment methods of cosmetic and functional outcomes in most published studies were thought to be prone to bias, subjectivity, or inaccuracy. Several assessment methods have been applied for evaluating cosmetic outcomes after TIP repairs, such as HOSE, PPPS, and HOPE [7–9]. However, all of them are retrospectively assessed, and which method is the most reliable and valid to assess the outcome is uncertain. From the practical point of view, it is highly recommended that standardized assessment tools be used for comparability and reproducibility and to build up a prospective database that does not currently exist. Moreover, there are many measurement indexes applied for the assessment of functional outcome in TIP repair, including urinary stream direction, maximum flow rate, voided volume, average flow rate, voiding time, flow curve shape, and residual urine. However, the significance of these measurement indexes remains uncertain until long-term follow-up studies clarify the significance of abnormal flow parameters [33]. Moreover, the assessment of functional outcomes in non-toilet-trained boys is difficult. Therefore, large prospective studies and uniform assessment criteria for functional and cosmetic evaluation are needed. Other outcomes, such as life quality, sexual function, and sexual psychology, are also not reported in any of the included studies.

DF is a layer of connective tissue found in the penile dorsal or ventral area, foreskin, and scrotum and can be used in hypospadias or fistula repair in different techniques [34, 35]. TVF can be harvested through a penile incision by degloving up to the root of the penis [26] or with an additional scrotal incision that reaches and covers the neourethra through a subcutaneous scrotal tunnel [36]. Excellent vascularity, easy availability and adequate source are advantages of DF, making this flap technique more popular for many paediatric urologists, especially young surgeons. Penile rotation and preputial skin necrosis are commonly reported relevant complications in the use of DF and can be avoided by careful operation and technical improvement. However, harvest of TVF may damage the vas deferens or vessels of the testicles, resulting in scrotal abscess or scrotal haematoma, but were ultimately not reported in any of the included studies. Snodgrass described additional interposition of vascularized tissues between the tubularized plate and the glans closure dissected from the dorsal preputial and shaft skin [2]. Duckett has described that when dartos is separated from the skin, it compromises the vascularity of the overlying skin [37]. Thus, the dissection of DF may compromise the vascularity of the preputial skin covering and result in subsequent skin necrosis, which is consistent with the conclusion of our outcomes. The blood supply of the neourethra tissue may be affected due to the dissection and utilization of DF, which mainly comes from the shortage of dartos or preputial skin necrosis. Although skin necrosis was inconsequential in the long run, it did cause anxiety and distress to the families and resulted in more hospital visits. Moreover, dissection to raise DF may damage the intrinsic blood supply to the outer skin, which is transposed ventrally to provide skin cover and may consequently devitalize, leading to skin necrosis, and fistula formation. However, this is rarely affected in the TVF technique, as its ventral skin covering is almost never compromised. All of these factors can theoretically explain the advantage of TVF over DF. We performed this meta-analysis to prove the advantages of TVF over DF with the data.

The results of our meta-analysis were partially consistent with the results of a systematic review by Fahmy et al. [3]. However, there were several differences between the two studies. The study of Fahmy et al. included not only comparative studies but case series, which weakened the evidence. In addition, the literature retrieval process should be as comprehensive as possible, while there was only one database (PubMed) employed in his study. Our analysis included only comparative studies and searched 4 databases (PubMed, EMBASE, the Cochrane Library and Web of Science), a clinical trial register (clinicaltrials.gov) and several international meeting abstract archives. All of these sources enhanced our evidence.

There are several limitations in our meta-analysis. First, although a comprehensive retrieval was performed, only limited studies were included, and most included studies were nonrandomized clinical trials/studies without reporting prospective power calculations and non-inclusion of consecutive patients, which might bias the results. Given the diversity of types of included studies, the level of evidence for our findings is not high. Second, the duration of follow-up varied, ranging from 6 to 48 months. The relatively short length of follow-up limited the present study, as it is known that long-term follow-up is necessary to determine the true complication rate of hypospadias repair and Spinoit et al. [38] stated in their study that only 47.37% of complications appeared in the first year. Third, the definition of complications was inconsistent among all included studies which indicated that reporting complications also depended on different factors and a publication bias existed. A survey of North American paediatric urologists clearly showed that there is a discrepancy between complication rates reported in the literature and participants’ operative outcomes, regardless of practice setting, operative volume, or time in practice [39]. In addition, research aimed at studying the effects of different flaps on urethrocutaneous fistula postoperatively may result in bias in other complications. Last, other than follow-up periods and complications criteria, differences in other clinical characteristics, including study settings, patient age, thickness and width of flap, and hypospadias type existed, although the statistical heterogeneity was not high. The contribution of these differences to the outcomes was unknown. We performed subgroup analyses to identify potential sources of heterogeneity, but no significant results were found due to the limited number of studies.

Although many uncontrollable confounding factors may affect the hypospadias surgery outcomes, especially the wide variability for individual surgical experience and complexity for hypospadias cases, additional large sample size, well-designed, single-urologist prospective studies need to be conducted for optimal comparisons between these two flap techniques.

## Conclusions

This meta-analysis revealed that compared to DF, TVF is a better option in TIP repair in terms of decreasing the incidence of the total complications, urethrocutaneous fistula, and prepuce-related complications. There is limited evidence for functional and cosmetic outcomes. However, there was substantial heterogeneity between studies indicating a lack of clarity in this field. There remains a need for high-quality prospective comparative trials for reliable evidence and clinical utility.

## Supplementary information

**Additional file 1.** **Supplemental Table 1.** PRISMA Checklist. **Supplemental Table 2.** Down and Black scale and scores for included studies. **Supplemental Figure 1.** Sensitivity analysis by changing the model to a random-effect model. (**a**) total complications, (**b**) each complication. **Supplemental Figure 2.** Sensitivity analysis by omitting each study. (**a**) total complications, (**b**) urethrocutaneous fistula, (**c**) meatal/urethral stenosis, (**d**) wound-related complications, (**e**) prepuce-related complications.

## Data Availability

All data generated or analysed during this study are included in this published article and its supplementary information files.

## Abbreviations

TIP: Tubularized incised plate
DF: Dartos fascia
TVF: Tunica vaginalis fascia
RCT: Randomized controlled trials
RR: Risk ratio
CI: Confidence intervals
