# Supplementary material for: Comparison of effect between dartos fascia and tunica vaginalis fascia in TIP urethroplasty: a meta-analysis of comparative studies
Source: BMC Urol. 2020 Oct 15;20:161. doi: 10.1186/s12894-020-00737-9 (PMC7559339; doi:10.1186/s12894-020-00737-9)
Supplement: Supplementary file 1 — Additional file 1. Supplemental Table 1. PRISMA Checklist. Supplemental Table 2. Down and Black scale and scores for included studies. Supplemental Figure 1. Sensitivity analysis by changing the model to a random-effect model. (a) total complications, (b) each complication. Supplemental Figure 2. Sensitivity analysis by omitting each study. (a) total complications, (b) urethrocutaneous fistula, (c) meatal/urethral stenosis, (d) wound-related complications, (e) prepuce-related complications. [file 12894_2020_737_MOESM1_ESM.docx]

**Table S1.** PRISMA Checklist

| **Section/Topic** | **#** | **Checklist Item** | | **Reported on Page #** |
| --- | --- | --- | --- | --- |
| **TITLE** | | | | |
| Title | 1 | Identify the report as a systematic review, meta-analysis, or both. | | Page 1 |
| **ABSTRACT** | | | | |
| Structured summary | 2 | Provide a structured summary including, as applicable: background; objectives; data sources; study eligibility criteria, participants, and interventions; study appraisal and synthesis methods; results; limitations; conclusions and implications of key findings; systematic review registration number. | | Page 2 |
| **INTRODUCTION** | | | | |
| Rationale | 3 | Describe the rationale for the review in the context of what is already known. | | Page 4 |
| Objectives | 4 | Provide an explicit statement of questions being addressed with reference to participants, interventions, comparisons, outcomes, and study design (PICOS). | | Page 4 |
| **METHODS** | | | | |
| Protocol and registration | 5 | Indicate if a review protocol exists, if and where it can be accessed (e.g., Web address), and, if available, provide registration information including registration number. | | Page 4 |
| Eligibility criteria | 6 | Specify study characteristics (e.g., PICOS, length of follow-up) and report characteristics (e.g., years considered, language, publication status) used as criteria for eligibility, giving rationale. | | Page 5 |
| Information sources | 7 | Describe all information sources (e.g., databases with dates of coverage, contact with study authors to identify additional studies) in the search and date last searched. | | Page 4 |
| Search | 8 | Present full electronic search strategy for at least one database, including any limits used, such that it could be repeated. | | Page 5 |
| Study selection | 9 | State the process for selecting studies (i.e., screening, eligibility, included in systematic review, and, if applicable, included in the meta-analysis). | | Fig. 1 |
| Data collection process | 10 | Describe method of data extraction from reports (e.g., piloted forms, independently, in duplicate) and any processes for obtaining and confirming data from investigators. | | Page 6 |
| Data items | 11 | List and define all variables for which data were sought (e.g., PICOS, funding sources) and any assumptions and simplifications made. | | Page 5-6 |
| Risk of bias in individual studies | 12 | Describe methods used for assessing risk of bias of individual studies (including specification of whether this was done at the study or outcome level), and how this information is to be used in any data synthesis. | | Page 6 |
| Summary measures | 13 | State the principal summary measures (e.g., risk ratio, difference in means). | | Page 7 |
| Synthesis of results | 14 | Describe the methods of handling data and combining results of studies, if done, including measures of consistency (e.g., I^2^) for each meta-analysis. | | Page 7 |
| Risk of bias across studies | 15 | Specify any assessment of risk of bias that may affect the cumulative evidence (e.g., publication bias, selective reporting within studies). | Page 7 | |
| Additional analyses | 16 | Describe methods of additional analyses (e.g., sensitivity or subgroup analyses, meta-regression), if done, indicating which were pre-specified. | Page 7 | |
| **RESULTS** | | | | |
| Study selection | 17 | Give numbers of studies screened, assessed for eligibility, and included in the review, with reasons for exclusions at each stage, ideally with a flow diagram. | Fig. 1 | |
| Study characteristics | 18 | For each study, present characteristics for which data were extracted (e.g., study size, PICOS, follow-up period) and provide the citations. | Table 1 | |
| Risk of bias within studies | 19 | Present data on risk of bias of each study and, if available, any outcome level assessment (see item 12). | Table 1 | |
| Results of individual studies | 20 | For all outcomes considered (benefits or harms), present, for each study: (a) simple summary data for each intervention group (b) effect estimates and confidence intervals, ideally with a forest plot. | Page 7-8 | |
| Synthesis of results | 21 | Present the main results of the review. If meta-analyses done, include for each, confidence intervals and measures of consistency. | Page 8-9 | |
| Risk of bias across studies | 22 | Present results of any assessment of risk of bias across studies (see Item 15). | Page 7 | |
| Additional analysis | 23 | Give results of additional analyses, if done (e.g., sensitivity or subgroup analyses, meta-regression [see Item 16]). | Page 8 | |
| **DISCUSSION** | | | | |
| Summary of evidence | 24 | Summarize the main findings including the strength of evidence for each main outcome; consider their relevance to key groups (e.g., healthcare providers, users, and policy makers). | Page 9 | |
| Limitations | 25 | Discuss limitations at study and outcome level (e.g., risk of bias), and at review-level (e.g., incomplete retrieval of identified research, reporting bias). | Page 12 | |
| Conclusions | 26 | Provide a general interpretation of the results in the context of other evidence, and implications for future research. | Page 13 | |
| **FUNDING** | | | | |
| Funding | 27 | Describe sources of funding for the systematic review and other support (e.g., supply of data); role of funders for the systematic review. | Page 14 | |

*From:* Moher D, Liberati A, Tetzlaff J, Altman DG, The PRISMA Group (2009). Preferred Reporting Items for Systematic Reviews and Meta-Analyses: The PRISMA Statement. PLoS Med 6(6): e1000097. doi:10.1371/journal.pmed1000097

**Table S2. Down and Black scale and scores for included studies**

**Table S2-1. Down and Black scores for included studies by Rater 1**

| Study | Reporting | External validity | Internal validity- bias | Internal validity-confounding | Power | Total scores |
| --- | --- | --- | --- | --- | --- | --- |
| Babu 2013 | 9 | 3 | 4 | 3 | 2 | 21 |
| Basavaraju 2017 | 8 | 3 | 3 | 3 | 2 | 19 |
| Chatterjee 2004 | 6 | 3 | 3 | 3 | 1 | 16 |
| Dhua 2012 | 10 | 3 | 5 | 5 | 1 | 24 |
| Gajbhiye 2018 | 8 | 3 | 3 | 3 | 1 | 18 |
| Kurbet 2018 | 10 | 3 | 6 | 5 | 1 | 25 |

**Table S2-2. Down and Black scores for included studies by Rater 2**

| Study | Reporting | External validity | Internal validity- bias | Internal validity-confounding | Power | Total scores |
| --- | --- | --- | --- | --- | --- | --- |
| Babu 2013 | 9 | 3 | 4 | 3 | 2 | 21 |
| Basavaraju 2017 | 8 | 3 | 1 | 3 | 2 | 17 |
| Chatterjee 2004 | 6 | 3 | 4 | 2 | 1 | 16 |
| Dhua 2012 | 10 | 3 | 5 | 4 | 1 | 23 |
| Gajbhiye 2018 | 8 | 3 | 3 | 3 | 2 | 19 |
| Kurbet 2018 | 10 | 3 | 6 | 5 | 2 | 26 |

**Table S2-3. Total Down and Black scores for included studies and Kappa Statistic**

| Study | Rater 1 | Rater 2 | mean | Kappa value | *P* |
| --- | --- | --- | --- | --- | --- |
| Babu 2013 | 21 | 21 | 21 | 0.571 | 0.121 |
| Basavaraju 2017 | 19 | 17 | 18 |  |  |
| Chatterjee 2004 | 16 | 16 | 16 |  |  |
| Dhua 2012 | 24 | 23 | 23.5 |  |  |
| Gajbhiye 2018 | 18 | 19 | 18.5 |  |  |
| Kurbet 2018 | 25 | 26 | 25.5 |  |  |

For total Down and Black scores, <16 was regarded as high-risk, 16-23 was regarded as moderate-risk, 24-32 was regarded as high-risk.

***Down and Black scale:***

***Reporting***

1. *Is the hypothesis/aim/objective of the study clearly described? (score: 0/1)*

2. *Are the main outcomes to be measured clearly described in the Introduction or Methods section? (0/1)*

3. *Are the characteristics of the patients included in the study clearly described? (0/1)*

4. *Are the interventions of interest clearly described? (0/1)*

5. *Are the distributions of principal confounders in each group of subjects to be compared clearly described? (0/1/2)*

6. *Are the main findings of the study clearly described? (0/1)*

7. *Does the study provide estimates of the random variability in the data for the main outcomes? (0/1)*

8. *Have all important adverse events that may be a consequence of the intervention been reported? (0/1)*

9. *Have the characteristics of patients lost to follow-up been described? (0/1)*

10. *Have actual probability values been reported (e.g. 0.035 rather than <0.05) for the main outcomes except where the probability value is less than 0.001? (0/1)*

***External validity***

11. *Were the subjects asked to participate in the study representative of the entire population from which they were recruited? (0/1)*

12. *Were those subjects who were prepared to participate representative of the entire population from which they were recruited? (0/1)*

13. *Were the staff, places, and facilities where the patients were treated, representative of the treatment the majority of patients receive? (0/1)*

***Internal validity - bias***

14. *Was an attempt made to blind study subjects to the intervention they have received? (0/1)*

15. *Was an attempt made to blind those measuring the main outcomes of the intervention? (0/1)*

16. *If any of the results of the study were based on “data dredging”, was this made clear? (0/1)*

17. *In trials and cohort studies, do the analyses adjust for different lengths of follow-up of patients, or in case-control studies, is the time period between the intervention and outcome the same for cases and controls? (0/1)*

18. *Were the statistical tests used to assess the main outcomes appropriate? (0/1)*

19. *Was compliance with the intervention/s reliable? (0/1)*

20. *Were the main outcome measures used accurate (valid and reliable)? (0/1)*

***Internal validity - confounding (selection bias)***

21. *Were the patients in different intervention groups (trials and cohort studies) or were the cases and controls (case-control studies) recruited from the same population? (0/1)*

22. *Were study subjects in different intervention groups (trials and cohort studies) or were the cases and controls (case-control studies) recruited over the same period of time? (0/1)*

23. *Were study subjects randomised to intervention groups? (0/1)*

24. *Was the randomised intervention assignment concealed from both patients and health care staff until recruitment was complete and irrevocable? (0/1)*

25. *Was there adequate adjustment for confounding in the analyses from which the main findings were drawn? (0/1)*

26. *Were losses of patients to follow-up taken into account? (0/1)*

***Power***

27. *Did the study have sufficient power to detect a clinically important effect where the probability value for a difference being due to chance is less than 5%? (0/1/2/3/4/5)*


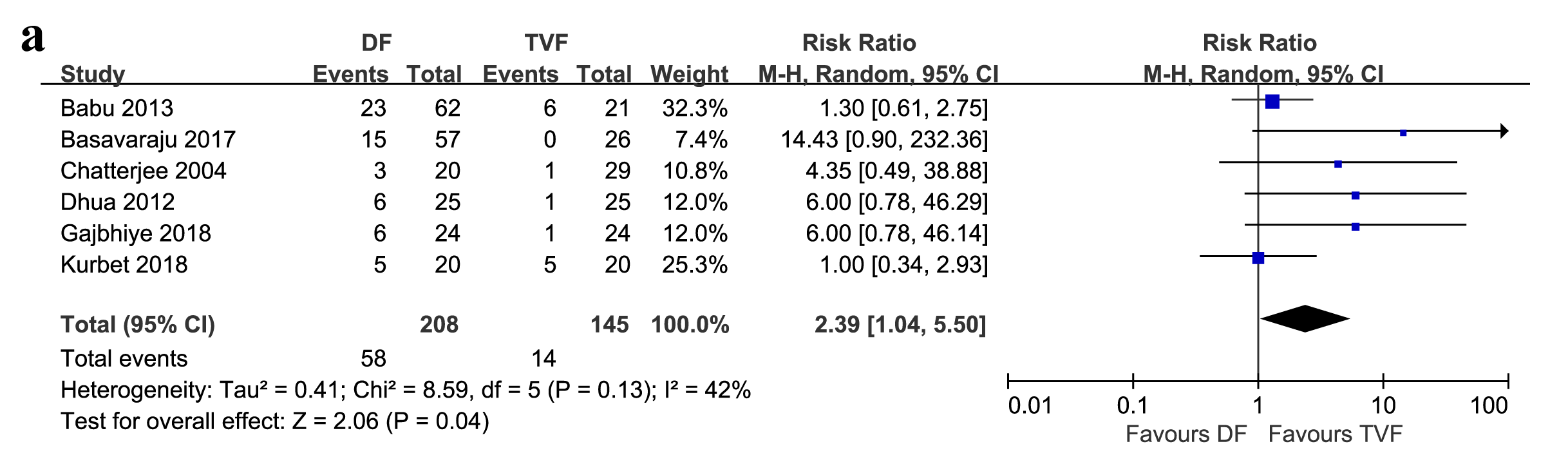


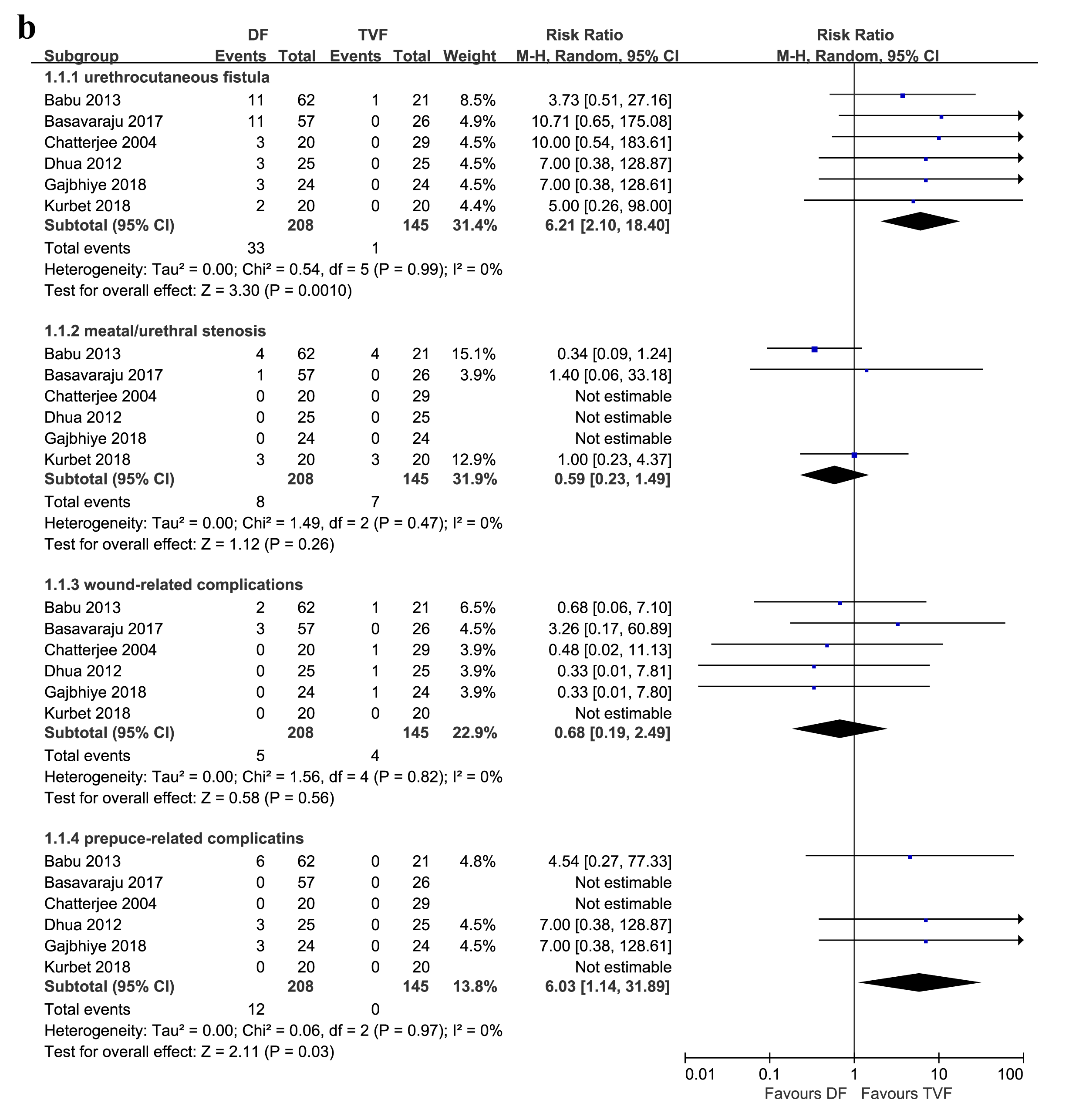


**Figure S1.** Sensitivity analysis by changing the model to a random-effect model. (**a**) total complications, (**b**) each complication

DF: dartos fascia; TVF: tunica vaginalis fascia

**a**

**b**

**c**

**d**

**e**

**Figure S2.** Sensitivity analysis by omitting each study. (**a**) total complications, (**b**) urethrocutaneous fistula, (**c**) meatal/urethral stenosis, (**d**) wound-related complications, (**e**) prepuce-related complications
